# Structure of the Core Postfusion Porcine Endogenous Retrovirus Fusion Protein

**DOI:** 10.1128/mbio.02920-21

**Published:** 2022-01-25

**Authors:** Trevor T. Dean, Vitor Hugo B. Serrão, Jeffrey E. Lee

**Affiliations:** a Department of Laboratory Medicine and Pathobiology, Temerty Faculty of Medicine, University of Toronto, Toronto, Ontario, Canada; Virginia Polytechnic Institute and State University

**Keywords:** endogenous retrovirus, porcine endogenous retrovirus, fusion protein, structural alignment, fusogen, membrane fusion, retroviruses, viral entry, viral glycoprotein

## Abstract

Retroviral elements from endogenous retroviruses have functions in mammalian physiology. The best-known examples are the envelope proteins that function in placenta development and immune suppression. Porcine endogenous retroviruses (PERVs) are an understudied class of endogenous retroviruses that infect cultured human cells, raising concern regarding porcine xenografts. The PERV envelope glycoprotein has also been proposed as a possible swine syncytin with a role in placental development. Despite the growing interest in PERVs, their envelope glycoproteins remain poorly characterized. Here, we successfully determined the postfusion crystal structure of the PERV core fusion ectodomain. The PERV fusion protein structure reveals a conserved class I viral fusion protein six-helix bundle. Biophysical experiments demonstrated that the thermodynamic stability of the PERV fusion protein secondary structure was the same at physiological and acidic pHs. A conserved surface analysis highlights the high degree of sequence conservation among retroviral fusogens in the chain reversal region that facilitates the large-scale conformational change required for membrane fusion. Further structural alignment of class I viral fusogens revealed a phylogenetic clustering that shows evolution into various lineages that correlate with virus mechanisms of cell entry. Our work indicates that structural dendrograms can be used to qualitatively infer insights into the fusion mechanisms of newly discovered class I viral fusogen structures.

## INTRODUCTION

Retroviruses are a unique family of viruses with a life cycle that circumvents the central dogma of molecular biology. The reverse transcription of the retroviral RNA genome and subsequent integration of resulting DNA within the host genome can disrupt host gene expression or protein translation (1). Another long-term consequence of the integration of the viral genome is endogenization, the process through which germ cells that have acquired viral DNA are part of a successful fertilization event (2). The genome of the resulting individual contains viral DNA. It is estimated that 8% of the human genome is made up of ancient human endogenous retrovirus DNA (3). In most cases, endogenous retroviral elements accumulate mutations and epigenetic modifications to prevent the production of infectious viral particles (4, 5); however, there are instances where endogenous viral elements play significant roles in host physiology (6). For example, the long terminal repeats of the elements may contribute to tissue-specific gene expression, and endogenous viral envelope glycoproteins (GPs) have been linked to the development of specialized tissue in diverse mammalian placentae (6–8).

Another notable example of endogenous retroviruses is the porcine endogenous retroviruses (PERVs). PERVs belong to the γ-retrovirus genus of porcine type C oncoviruses. PERVs can be further divided into distinct subclasses, PERV-A, -B, -C, and -A/C. All PERV subclasses, except for PERV-C, are polytropic, meaning they can infect porcine and human cells (9, 10). Despite the differences in tropism, PERV envelope GPs (also known as Env) share strong sequence conservation, ranging from 71% to 84% sequence identity. The ability of PERVs to infect human cells has raised alarm in the xenotransplant community where porcine tissues and organs are being considered alternatives for transplantation (11). Porcine cells and organs have been shown to produce PERV particles, which presents a risk of zoonotic transmission if left to circulate in humans (12). Additionally, PERVs are considered to be a source of envelope GPs that may be leveraged in porcine placentogenesis (13). Although PERVs have received significant attention for their prospective role in xenotransplantation, there is little biochemical data regarding PERV envelope GPs.

Retroviral envelope GPs belong to the class I viral fusion protein family, which includes HIV-1 gp160, coronavirus spike S, and influenza hemagglutinins, among others (14, 15). The PERV GP forms a metastable prefusion trimer on the surface of the virus. Each protomer is composed of two subunits, a surface protein (SU) that harbors receptor binding activity and a transmembrane protein (TM) that facilitates membrane fusion. A triggering event results in a conformational change required for membrane fusion. In the case of γ-retroviral proteins, the two SU and TM subunits of the envelope protein protomer are linked by a disulfide bond. Research into human T-cell leukemia virus 1 (HTLV-1) membrane fusion demonstrated that following receptor binding, a disulfide isomerization event rearranges the intersubunit covalent linkage between SU and TM to trigger large-scale conformational changes in the TM subunit that result in formation of the energetically favorable postfusion six-helix bundle (6HB) conformation (16, 17). Various crystallographic structures have confirmed the presence of a conserved postfusion 6HB that is predominantly α-helical in nature (14). This structural rearrangement brings the host and virus membranes in proximity to allow formation of the hemifusion membrane intermediate and subsequent membrane-pore formation. These pores result in cytoplasmic mixing, permitting viral contents to be deposited within the host cell.

Endogenous retroviruses provide an opportunity to study the functional and structural evolution of retroviral proteins, as they represent ancient members of the retrovirus family. In the context of envelope GPs, the study of endogenous viral elements can reveal ancient virus-cell fusion mechanisms. Here, we present the first postfusion PERV TM ectodomain crystal structure at 2.0 Å resolution. The PERV TM structure was stable over a pH range of 4.5 to 7.5, albeit with a significantly lower melting temperature than the related exogenous γ-retrovirus, xenotropic murine leukemia virus-related virus (XMRV). Furthermore, a conserved surface analysis of retroviral TM proteins that use a pH-independent fusion mechanism revealed sequence conservation of a chain reversal region that plays a critical role in the conformational change from pre- to postfusion states. Structure-based alignment of class I viral fusion proteins from distinct viral families provides a more reliable means to study protein ancestry and evolution than classical sequence alignments due to the rapid mutation of viral genomes and provides an alternate approach to qualitatively assess novel fusion GP structures. Structural phylogenetic analysis of class I postfusion viral GPs revealed structural clusters that stratify based on the mechanism of viral entry and identified new evolutionary links.

## RESULTS AND DISCUSSION

### PERV TM ectodomain design, purification, and structure determination.

**FIG 1:**
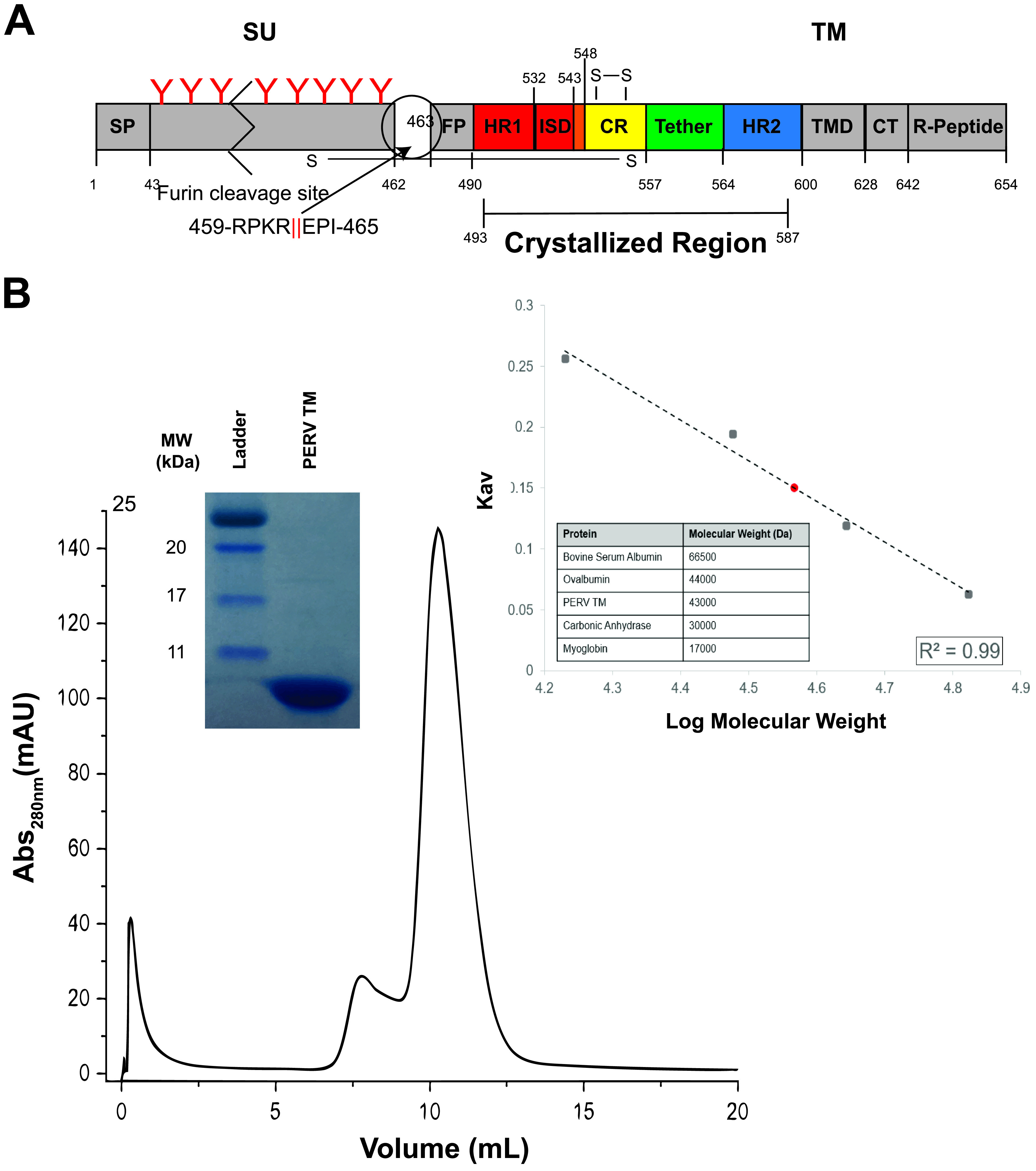
Postfusion PERV TM is a trimer in solution. (A) Overview of key regions of the PERV TM. The PERV envelope GP is translated as a single polypeptide that contains a signal peptide (SP) that allows the secretion and display of the GP on the membrane surface. The attachment subunit (SU) is N-linked glycosylated and is thought to be shed from the fusion subunit (TM) following receptor binding. The red “Y” symbols denote predicted N-linked glycans, and the zigzag indicates the SU is not drawn to scale. The TM fusogen contains the fusion peptide (FP) and the α-helical heptad repeats (HR1 and HR2) separated by a CX_6_CC-containing CR region and short six-residue tether. The PERV immunosuppressive domain (ISD) is present in the transition region from HR1 to the CR. A cysteine pair is formed between the first and second cysteine residues of the CX_6_CC motif, and the third cysteine makes an intermolecular disulfide bond with the SU. At the C-terminal end of the TM subunit are the transmembrane domain (TMD) and the cytoplasmic tail (CT), which contains the inhibitory R peptide. The crystallized region of the protein is indicated. (B) Size exclusion chromatogram of PERV TM_493–587_ on a Superdex 75 10/300 GL column. Purity of the peak fraction was assessed with a 16% Coomassie-stained gel (inset). A calibration *K*_av_ standard curve (inset) with bovine serum albumin (66,500 Da), ovalbumin (45,000 Da), carbonic anhydrase (30,000 Da), and myoglobin (17,000 Da) standards displayed as black squares was used to assess molecular weight. γ-Globulin (158,000 Da) was used to measure the void volume (*V_o_*). The apparent tag-intact PERV TM molecular weight was estimated to be ∼43 kDa (shown as a red circle), consistent with a trimeric biological assembly (theoretical trimeric MW, 39.9 kDa).

Secondary structural prediction of the full-length PERV envelope GP and location of the furin cleavage site was used to identify the boundaries of the SU and TM subunits. A construct that included the residues 493 to 587, encompassing the heptad repeat region 1 (HR1), the CX_6_CC chain reversal (CR) region, and HR2, was generated (Fig. 1A). The third cysteine in the CX_6_CC motif was mutated to serine (C557S) to prevent nonspecific disulfide formation from an unpaired cysteine. The equivalent third cysteine in other CX_6_CC-containing viral GPs, such as in Ebola virus (EBOV), Marburg virus (MARV), and HTLV-1, forms an intersubunit disulfide bond between the CR region to the SU subunit in the full-length protein (16, 17). There are no predicted N-linked glycans within the PERV TM ectodomain core.

**FIG 2:**
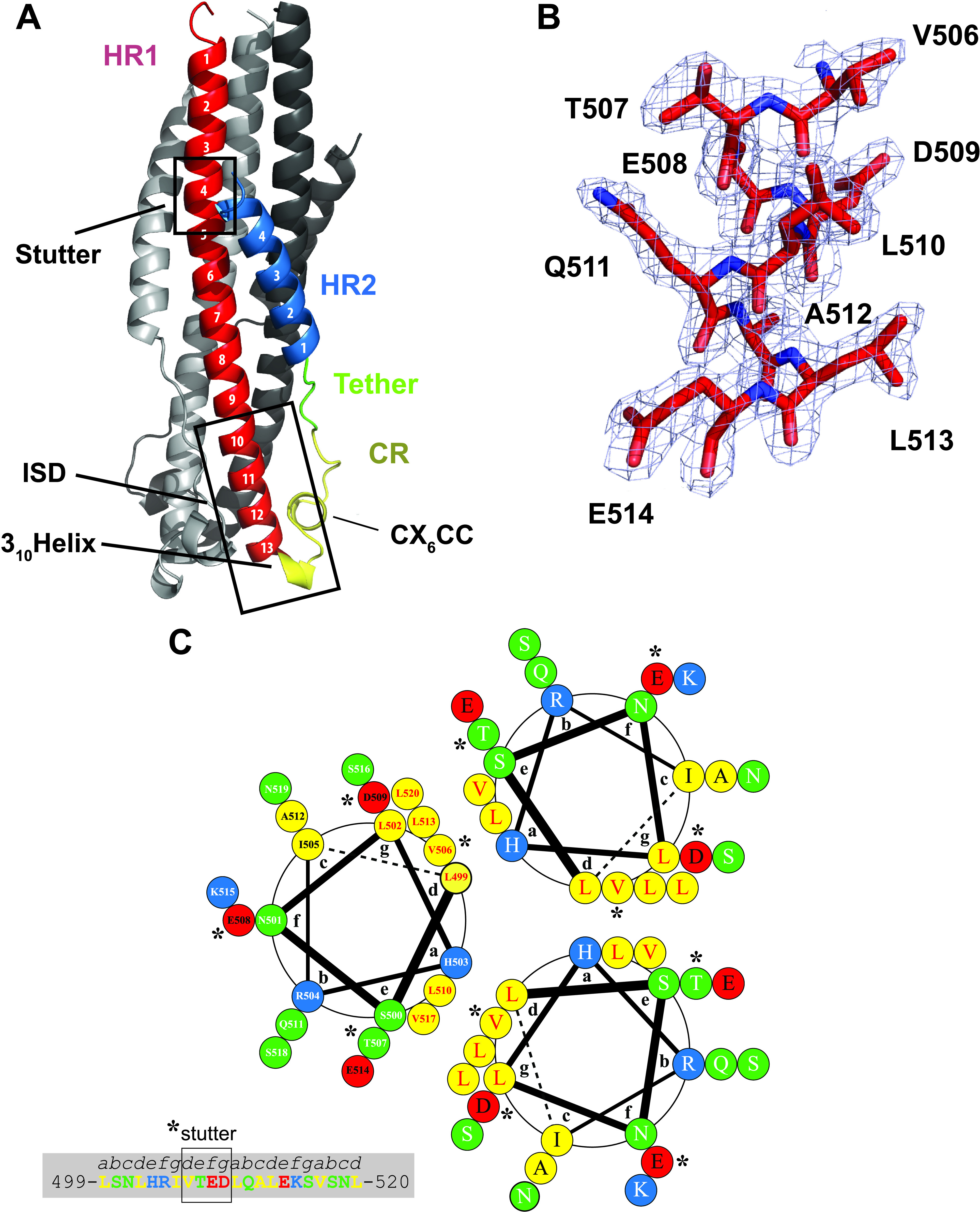
PERV TM displays a 6HB stabilized by electrostatic interactions. (A) Annotated ribbon representation of the PERV TM_493–587_ structure. The frontmost protomer is color-coded according to the schematic presented in Fig. 1A. Key functional and structural regions of the PERV TM are annotated. (B) Representative 2|*F*_o_| − |*F*_c_| electron density map contoured at 1σ and superimposed on the final refined PERV TM structure. Strong, well-defined electron density is observed throughout the HR1, CR, tether, and HR2 regions. (C) Helical wheel analysis of the central PERV HR1 trimer coiled-coil from residues 499 to 520 (helix turns 2 to 6) with the heptad repeat labeled a to g. Hydrophobic, polar, negatively charged and positively charged residues are colored yellow, green, red, and blue, respectively. Residue numbering is presented in one of the helical protomers. The presence of ^506^VTED^509^ in the PERV TM (highlighted with asterisks) results in a stutter of the heptad repeat, where it disrupts the periodicity of hydrophobic residues at the a and d positions of the heptad repeat.

The PERV TM_493–587_ ectodomain was heterologously expressed in Escherichia coli and purified by standard immobilized Ni-metal affinity and size exclusion chromatography (Fig. 1B). PERV TM_493–587_ (tag intact) eluted from the gel filtration column with an apparent molecular weight (MW) of ∼43 kDa, consistent with formation of a trimer in solution (theoretical trimeric MW, 39.9 kDa) (Fig. 1B). Other postfusion class I viral fusion proteins that have been biochemically and structurally characterized also form trimers (18, 19). The purified PERV TM ectodomain was crystallized, and its structure was determined by molecular replacement to 2.0 Å resolution (Fig. 2A). Two biological trimers are present in the asymmetric unit. The electron densities of both PERV biological trimers are well defined in the HR1, CR, tether, and HR2 regions (Fig. 2B).

### Overall structure of the PERV TM.

The PERV TM core is the first structure determined from the porcine endogenous retrovirus family. The PERV TM structural analysis revealed a canonical class I viral postfusion 6HB. Each protomer has two heptad-repeat regions (HR1 and HR2) separated by a CR region. The canonical trimeric 6HB is organized in coiled-coils, with the N-terminal HR1 forming the central trimeric helical core and the C-terminal HR2 packed against it (Fig. 2A). These characteristic HR regions form a seven-residue structural motif (a, b, c, d, e, f, g), where the first and fourth amino acids are hydrophobic (Fig. 2C). The N-terminal HR1 helix is composed of 13 turns with leucine and valine residues at positions a and d, respectively, along the helix. The leucine and valine residues pack in a knob-in-hole arrangement that likely stabilizes the central coiled-coil core. Asparagine residues (N534) from each protomer point into the inner helical core to coordinate a chloride ion. The chloride is located on the 3-fold symmetr*y* axis, as commonly observed in the crystal structures of other class I viral fusion proteins (18, 19). The HR2 helix spans four turns and fits into a groove between two HR1 helices from two protomers. The repeating hydrophobic residues from HR2 form hydrophobic interactions with residues from HR1.

The PERV TM structure revealed an HR-interrupting stutter corresponding to ^506^VTED^509^, visible after the third turn in HR1 (Fig. 2A and C). The stutter breaks the periodicity of the heptad repeat by adding an extra “defg” repeat into the motif (*abcdefg****defg****abcdefg*). The four-residue stutter identified on the postfusion PERV TM structure is also observed in the 6HB structures of Mason-Pfizer monkey virus (MPMV), XMRV, bovine leukemia virus (BLV), and human syncytin 1 and syncytin 2 (20). HR-disrupting stutters have been identified in the coiled-coil domains of several other nonretroviral class I fusion proteins, including those of EBOV and lymphocytic choriomeningitis virus (LCMV) (21). The PERV TM stutter sits two and four α-helical turns N-terminal to the stutters from the filo- and arenaviral GP2s, respectively. The interruption of the hydrophobic core induces a local unwinding of the superhelical coiled-coil in the two helical turns downstream of the stutter (22). As previously described, this local unwinding causes a deviation from ideal geometry that manifests in a helical twist (20). These four residues that disrupt the HR impart structural flexibility to the long coiled-coils that are otherwise rigid due to the knob-in-hole packing of the HRs (22).

The CR region (residues 543 to 557) is composed of a short 3_10_ helix, a single-turn α-helix that is contained within a conserved CX_6_CC motif (C549-C557), the C-terminal portion of the immunosuppressive domain (ISD) (residues 532 to 548), and a short six-residue flexible tether that joins the CR region to the HR2 helix (Fig. 2A). The CR region forms a fold-back of the chain to facilitate the antiparallel orientation of the HR1 and HR2 helices, thus positioning the N and C termini together. The conserved CX_6_CC motif forms an intramotif disulfide bond between the first and second cysteines. The third cysteine in the motif is proposed to form an intersubunit disulfide bond to covalently link the SU and the TM in the full-length PERV Env. In δ- and γ-retroviruses, a CXXC thiol motif in the SU subunit participates in a disulfide exchange mechanism with the CX_6_CC TM motif to facilitate fusion (16, 17). The C-terminal portion of the HR1 helix and the 3_10_ helix form the 17-residue ISD (Fig. 2A). This short motif is well conserved among retroviral TMs and has been shown to alter cytokine expression and lymphocyte proliferation (23, 24).

### Network of HR1-HR2 electrostatic interactions.

**FIG 3:**
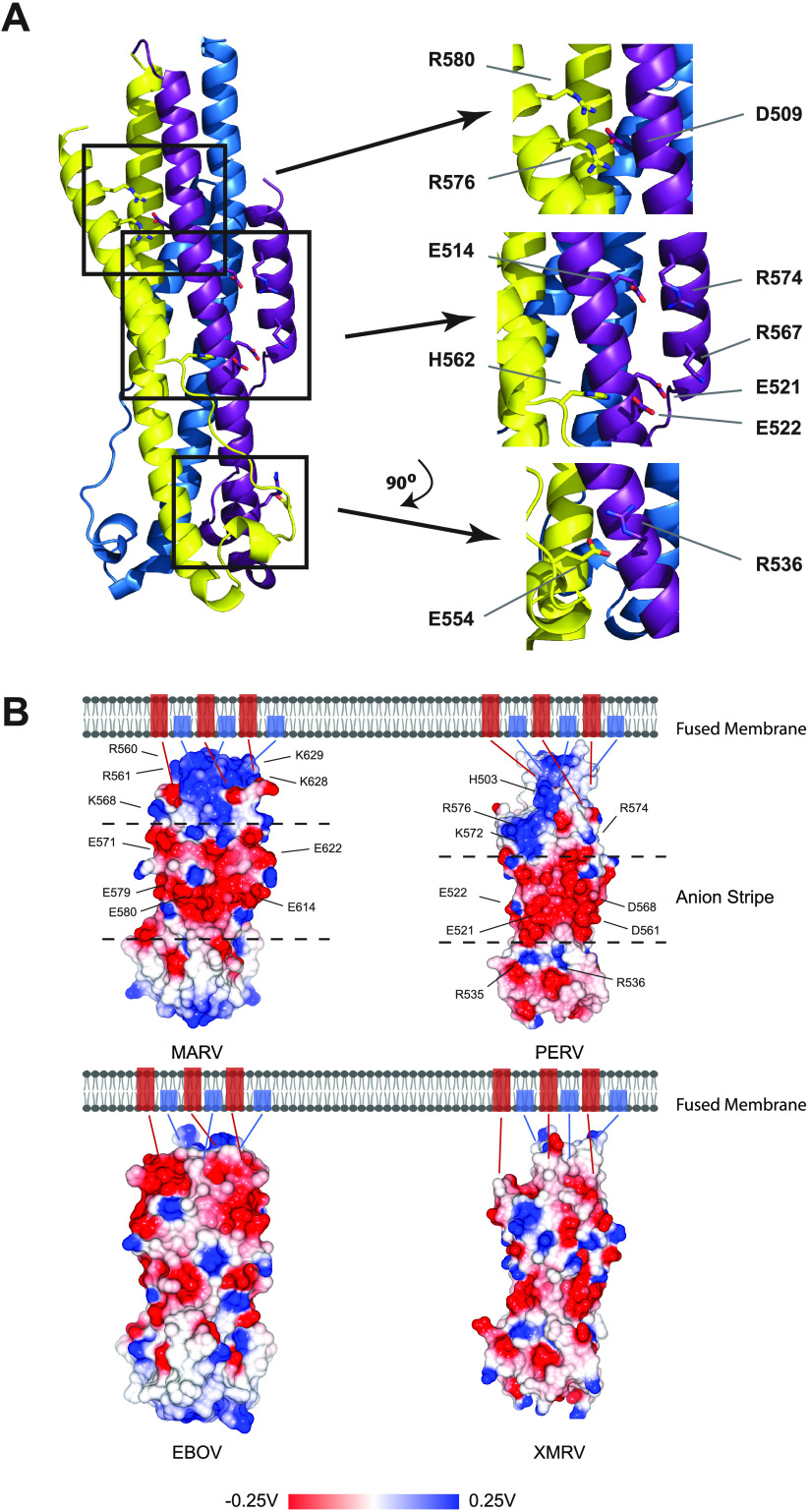
PERV TM structure displays a central anion stripe. (A) Ribbon representation of PERV TM intermolecular electrostatic cross-links. Three sets of complex intermolecular salt bridges are observed between protomers R576-D509-R580, E522-H562, and E554-R536. Residues E521 and E522 that contribute to the central anion stripe are indicated. (B) Surface electrostatic potential map calculated for postfusion EBOV, MARV, PERV, and XMRV TMs. Negatively charged anion stripes are displayed along the midsection of the MARV and PERV surfaces and are found between dashed lines that intersect the surfaces. Acidic residues that contribute to the anion stripe and basic residues that line the anion stripe are annotated. Fusion proteins without anions stripes from the same viral family are found on the bottom panel. Blue and red rectangles found in the membrane correspond to the N-terminal fusion peptide and C-terminal transmembrane domain, respectively. Electrostatic potential is given in units of volts.

The PERV 6HB is interlaced with a series of inter- and intrachain salt bridges (Fig. 3A). Three sets of electrostatic interactions are formed between protomers. A complex interchain salt bridge forms an electrostatic cross-link between HR1 (D509) and HR2 (R576 and R580) on a neighboring protomer. A second interchain salt bridge is formed between E522 on HR1 to H562 in the flexible tether from a neighboring unit. A third interchain salt bridge is formed in the CR region between E554 and R536 from a neighboring conserved retroviral “QNRR” motif. Two intrachain salt bridges are formed between HR1 and HR2 (E514-R574 and E521-R567). Notably, residues E521, E522, and D561 form an anion stripe along the midsection of the PERV 6HB (Fig. 3), similar to that reported for MARV GP2 (25). The anion stripe is sandwiched between regions that contain an increased density of basic residues (K572, R574, and R576 from HR2 above the stripe and R535 and R536 from HR1 below the stripe) (Fig. 3B). The MARV GP2 anion stripe is hypothesized to be responsible for the stability of the fusogen at acidic pH (25). MARV requires trafficking to the endosome where membrane fusion occurs at low pH (26). The anion stripe in MARV is critical for stabilizing the postfusion glycoprotein conformation at endosomal pH. On the other hand, PERV is expected to fuse on the cell surface at physiological pH; thus, it is not clear why the PERV TM displays an anion stripe. We can only speculate that given the structural similarities of the PERV TM and MARV GP2, these glycoproteins evolved from a common ancestor. The PERV TM anion stripe may be a vestigial feature left from this ancient ancestral glycoprotein that was lost in other retroviral fusion proteins over the course of evolution (Fig. 3B).

### PERV TM is thermally stable at low and high pH.

Research into the different retroviral genera revealed a link between the route of retroviral cell entry and fusion protein sensitivity to pH (18). Experiments contrasting the thermal stability of viral fusion proteins, which use pH-dependent fusion mechanisms, to those that use a pH-independent mechanism suggest that the stability of the fusion protein tends to mimic the environment where they fuse (18). For example, HTLV-1 TM, a retrovirus that fuses at the plasma membrane, is most stable at neutral pH (18). In contrast, EBOV, MARV, and influenza A virus (IAV), which fuse at the endosome, contain fusogens that are most stable at pH values lower than 5.5 (26–28). PERV envelope proteins are expected to facilitate virus-cell fusion on the cell surface at neutral pH like other members of the γ-retrovirus genus, such as XMRV. However, PERVs are ancient. They are estimated to be greater than 7 million years old (29), and thus, their function may differ from modern γ-retroviruses.

**FIG 4:**
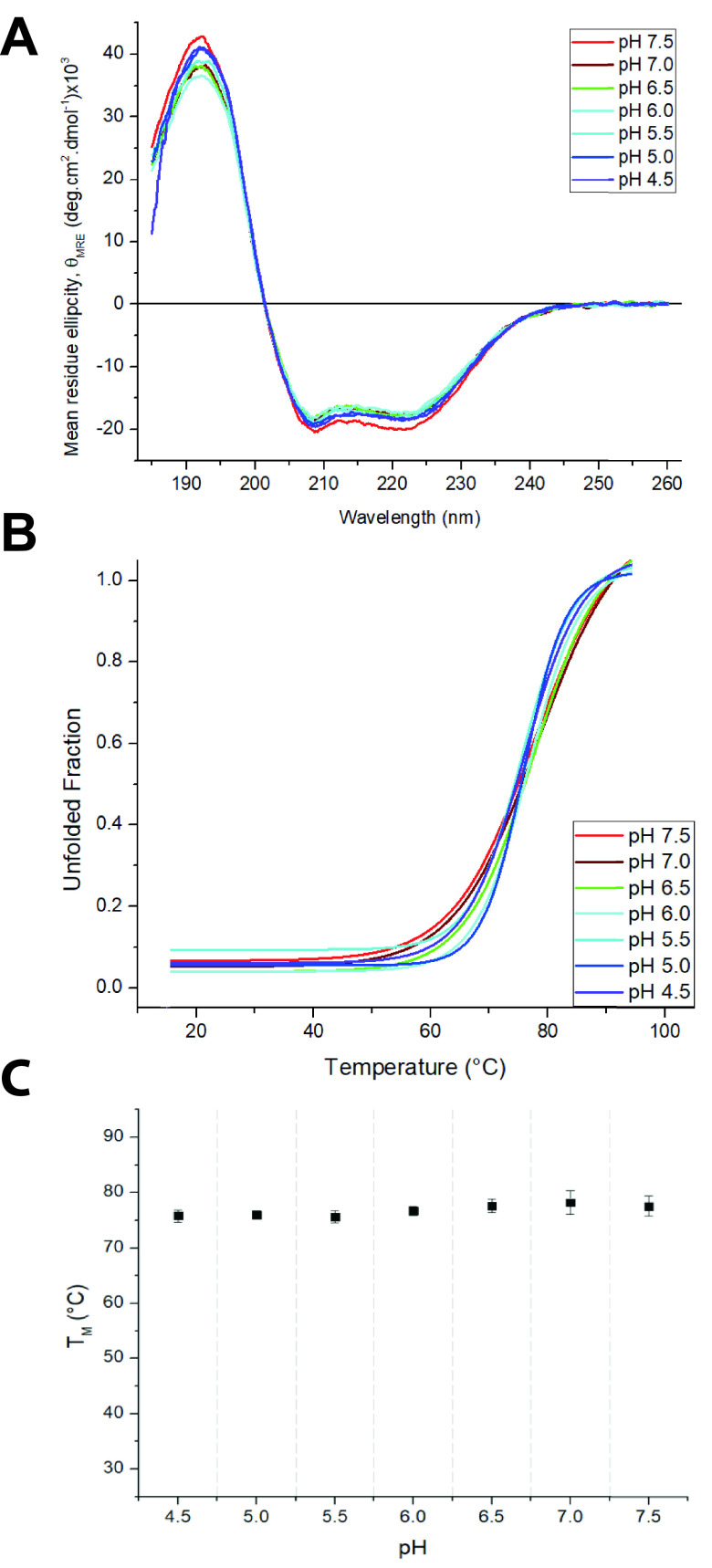
The thermodynamic stability of the PERV TM is independent of pH. (A) Superposition of PERV TM CD spectra at pH values ranging from 4.5 to 7.5. (B) Nonlinear biphasic sigmoidal curves fit to the thermal melt data to determine *T_m_* as a function of pH. Melting temperature errors were calculated based on curve fitting error. (C) Plot of *T_m_* versus pH. Error bars are standard errors from curve fitting.

To further characterize the PERV TM, we used circular dichroism (CD) spectroscopy to assess the stability of the 6HB ectodomain over a range of physiologically relevant pH values (pH 4.5 to 7.5). Secondary structure elements have distinct CD profiles that can provide insight into protein folding. Superimposition of the PERV TM CD spectra at various pH values revealed that the 6HB retained its helical structure at neutral and acidic pH values and no structural change is detected (Fig. 4A). Thermal denaturation experiments revealed that the PERV TM was equally stable at all the tested pHs (Fig. 4B and C). The stability of the PERV TM at acidic pHs starkly differs from thermal stabilities of other pH-independent retroviruses. For example, the melting temperature (*T_m_*) of the HTLV-1 TM is about 40°C lower at pH of <7.0 than at pH 7.0. Notably, the *T_m_* of the PERV TM was significantly lower (∼78°C) than the *T_m_* of the XMRV fusion protein, which required chemical destabilization to decrease the *T_m_* to less than 100°C (19). Rather, the PERV TM has a stability profile similar to the avian sarcoma leukosis virus (ASLV) TM, which undergoes a unique hybrid 2-step entry mechanism (18).

### CR region is strongly conserved among retroviral TMs that fuse at the plasma membrane.

**FIG 5:**
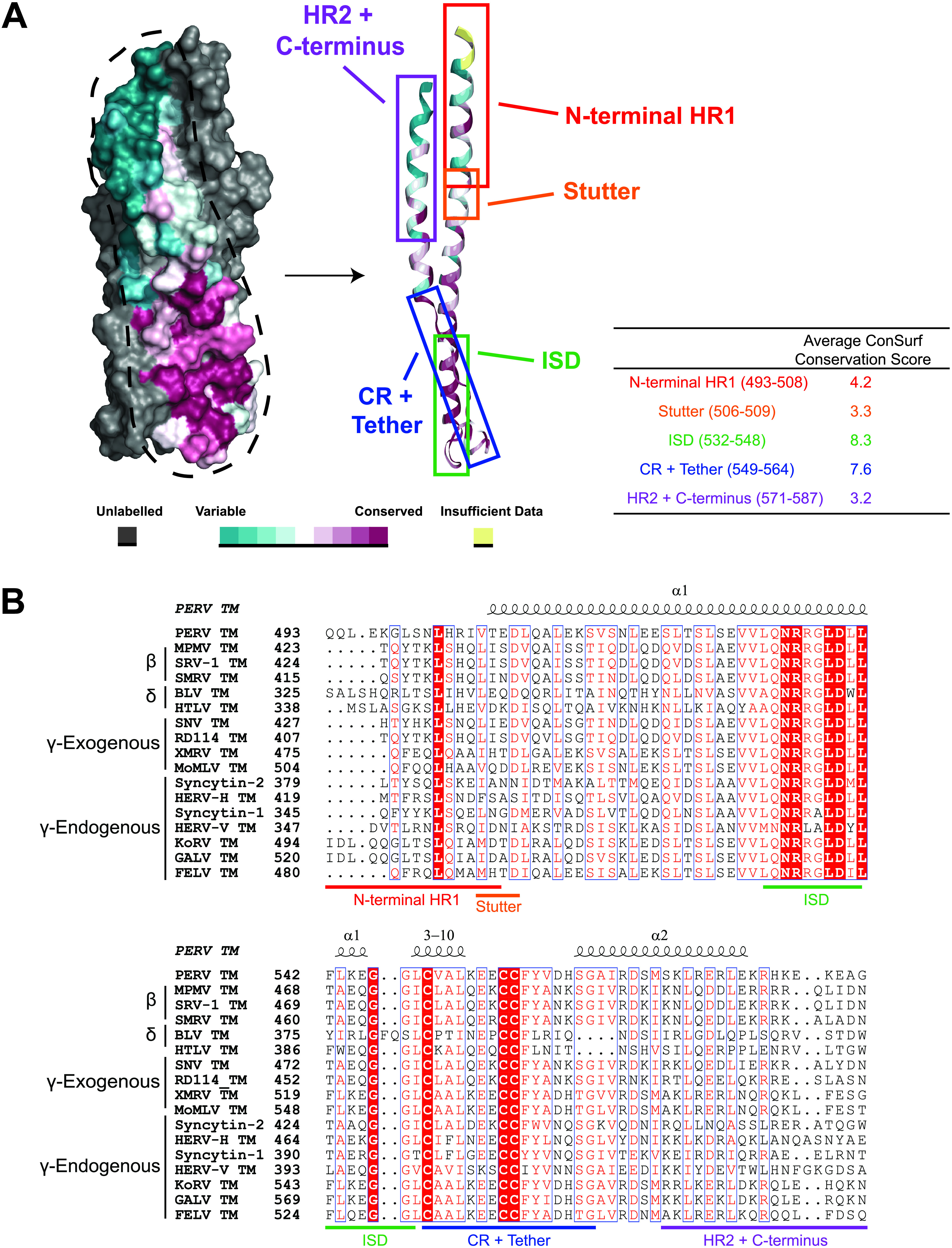
PERV TM has a conserved surface only in the chain reversal and tether regions. (A) Evolutionary rates determined from the retroviral TM sequence alignment were mapped onto the surface of the PERV TM structure. The protomer shown in front is colored according to a residue conservation gradient, and the two other chains are in gray. Conserved residues are colored in burgundy, and residues not conserved are shown in cyan. The table lists the average conservation scores of each region. (B) Multiple-sequence alignment of β-, δ-, γ-exogenous, and γ-endogenous retroviral glycoprotein sequences used to generate the conserved surface in panel A. The regions of the PERV TM used to determine the ConSurf conservation scores are highlighted below the multiple-sequence alignment, and the PERV TM secondary structural elements are displayed above. Glycoprotein sequence accession numbers are listed in Materials and Methods. PERV, porcine endogenous retrovirus; MPMV, Mason-Pfizer monkey virus; SRV-1, simian retrovirus 1; SMRV, squirrel monkey retrovirus; BLV, bovine leukemia virus; HTLV, human T-lymphotropic virus 1; SNV, spleen necrosis virus; RD114, RD114 retrovirus; XMRV, xenotropic murine leukemia virus-related virus; MoMLV, Moloney murine leukemia virus; HERV-H, human endogenous retrovirus H; HERV-V, human endogenous retrovirus V; KoRV, koala retrovirus; GALV, Gibbon ape leukemia virus; FELV, feline leukemia virus.

The retention of ancient retroviral gene products in mammalian genomes provides a unique opportunity to study how viral proteins have evolved over time. To study the evolution of retroviral fusogens, we analyzed envelope proteins from retroviruses that perform membrane fusion at the cell surface (fusion proteins from the β-, δ-, and γ-retroviral genera). For this evaluation, we selected envelope protein sequences from ancient endogenous retroviruses as well as modern exogenous retroviruses. Multiple-sequence alignments have demonstrated that retroviral envelope proteins have considerable sequence variability despite their common ancestry (19). Despite this, these proteins share a fusion mechanism and postfusion 6HB conformation. To further understand the conservation of retroviral fusogens, we performed a conserved surface (ConSurf) analysis to identify functional regions (30). The evolutionary rate of each amino acid is expected to be inversely correlated with functional and structural importance (31). The mapping of conservation scores onto the surface of the PERV TM structure revealed that the highest degree of sequence conservation is within the CR-tether and the ISD regions (Fig. 5A and B). The conservation of sequences in the CR-tether and ISD regions is suggestive of important functional roles for the sequences in these regions. The CR and tether motifs are critical to the protein dynamics required for membrane fusion, as these regions facilitate the transition from the prefusion to the postfusion 6HB conformation. Notably, the portion of the ISD with the greatest amount of sequence variability corresponds to the 3_10_ helix located at the end of HR1. Despite the divergence of the 3_10_ helix sequence, the ISD structural motif is well conserved among the β-, δ-, and γ-retroviral fusion protein structures solved to date. The sequences of the remainder of the CR and tether region (residues 549 to 564) have a high degree of similarity, with only conservative substitutions occurring within the single-turn α-helix and tether. Taken together, these results could indicate that the physicochemical properties and secondary structure motifs in the CR-tether are critical in the conformational change that facilitates membrane fusion. In contrast, there is significant sequence variability in the N-terminal half of HR1 and throughout HR2. The N- and C-terminal helices are likely more susceptible to mutation, as the interaction interface between HR1 and HR2 is mediated through more nonspecific hydrophobic interactions.

### Structural phylogenetic analysis identifies evolutionary lineages that are reflective of entry mechanism.

New viruses are continually being discovered, expanding the landscape of the virosphere (32), and analyses of endogenous retroviruses are also shedding light on the genetic diversity of viruses. The genetic diversity of viruses is vast; however, the diversity of surface viral GP structures is limited, as common protein folds and core structures have been identified from genetically distinct viral families (33–35). Identification of common folds and core structures provides the opportunity to establish structure-function links and insights into evolutionary connections between biologically and genetically divergent viruses. Evolutionary relationships and common ancestry may be difficult to infer using only the primary sequences of viral proteins, as viruses mutate about 1 million times more frequently than cellular organisms (36), and it is common that sequence similarities are low in functionally related proteins. An alternate approach is to compare the three-dimensional structures of viral proteins to identify conserved features and to establish new or modify existing classifications.

We used a structure-based method to determine how the PERV fusion subunit is related to other class I viral GPs. Over the past 3 decades, multiple postfusion structures of class I viral fusogens from various viral families have been deposited in the Protein Data Bank. We analyzed class I viral fusion proteins from a range of viral families with different entry mechanisms for which structures with resolutions 4.5 Å or better that contained intact HR1, HR2, and CR regions were available. Structural alignment of fusion proteins is complicated by the fact that class I fusogens vary across viral taxa. It was previously demonstrated that a conserved stutter found in HR1 of class I viral fusion GPs provides an alignment point that can be leveraged in the structural comparison of distinct fusogens (21). Thus, prior to structural alignments, we truncated the N termini of the HR1 so that all proteins started at the stutter.

**FIG 6:**
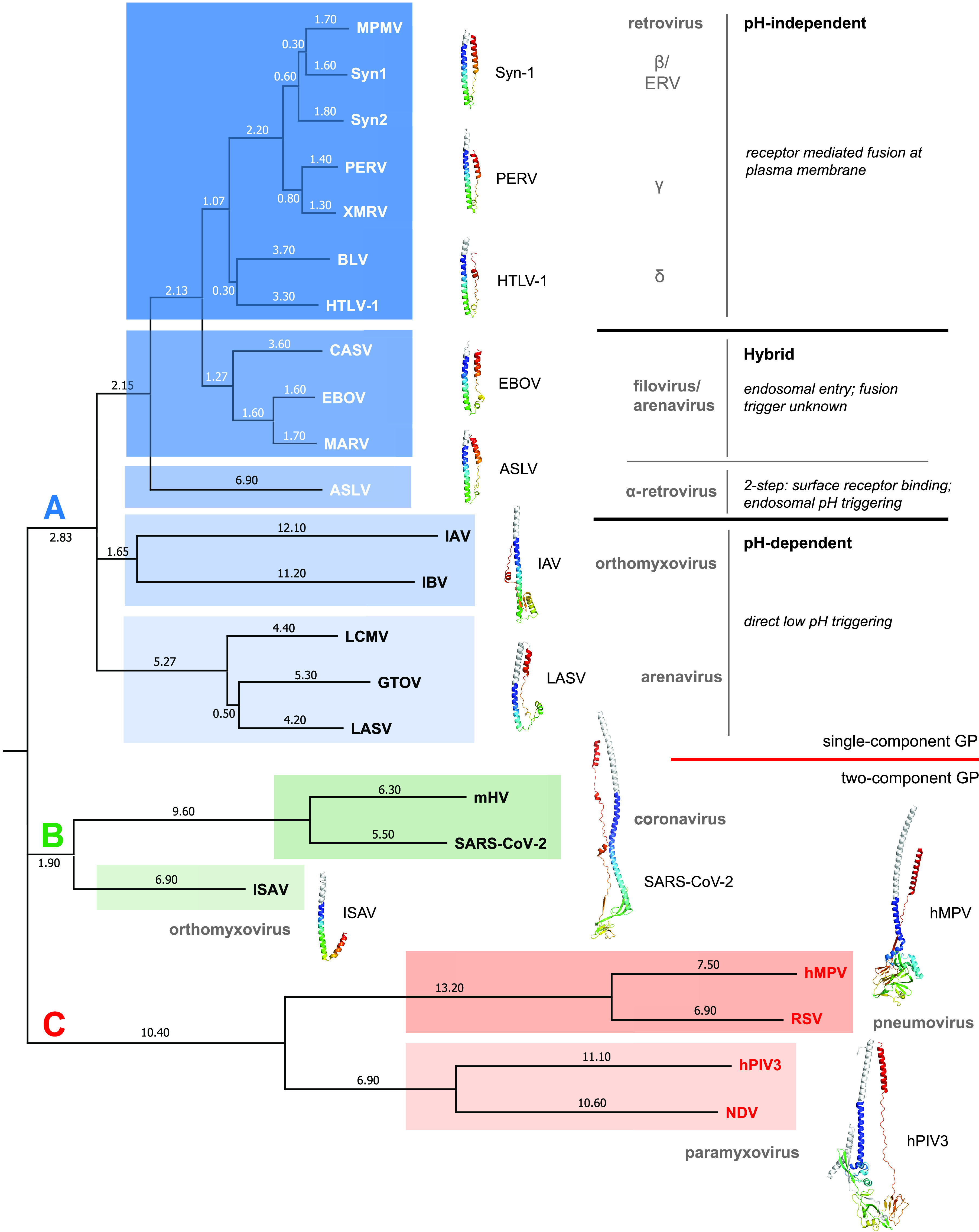
Structural dendrogram of class I postfusion subunits reveals clustering based on mechanism of viral entry. A structural dendrogram displaying the relationship between select class I viral postfusion structures as calculated by DALI (75–77). The evolutionary distances between structures are defined as the difference between the Z-score assigned to a structural pair and the sum of the Z-scores assigned to the self-alignment of each structure. Calculated evolutionary distances derived from the structural phylogenetic analysis are shown on the branch lines. Structures of representative viral fusion proteins from each family are displayed adjacent to the dendrogram. The distinct lineages are boxed and labeled in color. Viral classifications and fusion entry/triggering mechanisms are also annotated. Viruses are abbreviated as follows: ASLV, avian sarcoma leukosis virus; BLV, bovine leukemia virus; CASV, California Academy of Science virus; EBOV, Ebola virus; GTOV, Guanarito virus; hMV, human metapneumovirus; hPIV3, human parainfluenza virus 3; HTLV-1, human T-lymphotropic virus 1; IAV, influenza A virus; IBV, influenza B virus; ISAV, infectious salmon anemia virus; LASV, Lassa virus; LCMV, lymphocytic choriomeningitis virus; MARV, Marburg virus; MPMV, Mason-Pfizer monkey virus; mHV, murine hepatitis virus; NDV, Newcastle disease virus; RSV, respiratory syncytial virus; SARS-CoV-2, severe acute respiratory syndrome coronavirus-2; Syn-1, syncytin 1; Syn-2, syncytin 2; and XMRV, xenotropic murine leukemia virus-related virus.

Our structural phylogenetic analysis of class I viral fusion protein protomers revealed three unique lineages, A, B, and C (Fig. 6). A closer analysis showed that the region between the HR1 and HR2 is highly diverse and likely the major determinant of lineages in the structural phylogenetic tree. The diversity of the CR region was observed in previous studies using stutter-based alignments of class I viral fusogens (21). In general, lineages A, B, and C are separated based on the size of the CR region. For example, lineage C is composed of examples from the paramyxoviral (human parainfluenza virus 3 (hPIV3) and Newcastle disease virus (NDV) and pneumoviral (human metapneumovirus (hMPV) and respiratory syncytial virus (RSV) families of viral fusogens. CR regions of viruses in this lineage have more than 200 residues between the HR1 and HR2 helices. In contrast, lineage B, which contains the orthomyxoviral infectious salmon anemia virus (ISAV) F2, and the coronaviral mouse hepatitis virus (mHV) and severe acute respiratory syndrome coronavirus 2 (SARS-CoV-2) S2 proteins, display more varied CR lengths. Lineage A has CR regions that are of smaller size: the arenaviral GP2 proteins that facilitate membrane fusion at acidic pH have protruding 45-residue linkers, or T-loops, which contain conserved secondary structure elements and an intrachain disulfide bond. The two pH-dependent orthomyxovirus hemagglutinin (HA) proteins contain random-coil linkers that are about 10 residues in length, and both retro- and filoviral fusion proteins have 20-residue CR regions. As more structures of class I viral fusion proteins are determined with an intact CR region, the structural phylogenetic dendrogram will become more complete, and new associations will be discovered.

The structural dendrogram we constructed interestingly stratifies based on the mechanism of viral entry. Lineage A contains diverse viral GPs from the *Retroviridae*, *Arenaviridae*, *Orthomyxoviridae*, and *Filoviridae* families that were further stratified into distinct sublineages. These sublineage divisions were correlated with different mechanisms of viral entry and membrane fusion. For example, the PERV TM is grouped with other CX_6_CC-containing fusion proteins from viruses that fuse at the plasma membrane in a pH-independent manner. It clusters closest with the γ-retroviral fusion protein from XMRV, and this sublineage also includes the β-retroviral TMs from Mason-Pfizer monkey virus (MPMV) and human syncytins. This would be consistent, as PERV TM is suggested to catalyze fusion at the plasma membrane (37). These results suggest that the retroviral TMs that undergo fusion at the plasma membrane share a conserved fusion protein template. It also appears that endogenous retroviral TMs, such as PERV TM and syncytins, cluster with their exogenous retroviral relatives despite millions of years of evolution, suggesting the structural determinants to catalyze viral-cell and cell-cell membrane fusion are similar.

On the other hand, the α-retrovirus TMs, such as that from ASLV, represent a unique sublineage that is separate from other examples in lineage A. This is not surprising, as ASLV has a unique hybrid two-step entry mechanism that requires first binding to a host receptor at the plasma membrane in a pH-independent manner to trigger initial conformational changes to the ASLV Env. This is then followed by a second low-pH-induced refolding event in the endosome to complete the fusion reaction (38–41). The ASLV TM is different than the pH-dependent orthomyxovirus family hemagglutinin fusion proteins (HA2) from IAV and influenza B virus (IBV), which cluster into a separate sublineage. The conformational changes that IAV and IBV HA undergo during fusion are controlled solely by low pH.

Unexpectedly, the arenaviruses were stratified into two different sublineages, suggesting different evolutionary paths. Guarnarito virus (GTOV), Lassa virus (LASV), and LCMV proteins from New and Old World arenaviruses cluster together, suggesting a common structural framework; however, the fusogen from another arenavirus, the California Academia of Sciences virus (CASV) GP2, is in the sublineage with EBOV and MARV GP2s. The linker region of the CASV GP2 is similar in structure to those from Ebola and Marburg viruses (42). These data suggest that the reptarenavirus fusogen has a common ancestor in line with the filoviruses but differs from its mammarenavirus (GTOV, LASV, and LCMV) counterparts. The clustering of CASV, EBOV, and MARV GP2s is consistent from a mechanistic perspective. EBOV and MARV are endocytosed and trafficked to the endosome, where its GP is cleaved by host cathepsins, followed by binding to the endosomal Niemann-Pick C1 (NPC1) cholesterol transporter (43–47). While the entry pathway of CASV is not completely understood, endosomal inhibitors revealed a low-pH dependence for virus entry, similar to filoviruses (42). Thus, the CASV, EBOV, and MARV GP2 all need to be stable in a low-pH environment. However, low pH and/or receptor binding do not appear to trigger fusion for EBOV and MARV (48). This contrasts with LASV and LCMV glycoprotein complex (GPC), where their glycoproteins appear to be directly triggered by endosomal acidification (49). LASV and LCMV GPCs are also unique among the class I viral fusogens, as they form a heterotrimeric entry complex that contains the attachment, fusion, and stable signal peptide subunits (GP1-GP2-SSP complex) (50–52). Thus, the structural conformations of the fusion subunit suggest that the mammarenaviruses form a separate sublineage and have a unique mechanistic variation on fusion.

In contrast to lineage A, where a single GP is involved in attachment and fusion, lineages B and C consist of viruses that often have their attachment and fusion machinery on separate proteins. Paramyxoviruses, pneumoviruses, and orthomyxoviruses have two-component viral GP entry complexes with their fusion proteins still classified as class I viral GPs. For example, pneumoviral and paramyxoviral families in lineage C have two distinct GPs for attachment (HN, H, or G) and fusion (F) (53–57). In lineage B, ISAV is a piscine orthomyxovirus that has a hemagglutinin-esterase (HE) involved in attachment and receptor destruction and a separate F protein for fusion (58–61). Despite the relationship of ISAV to IAV and IAB, the structural dendrogram shows its fusion protein clustering into a distinct lineage from other orthomyxovirus HA2s (lineage A), suggesting a different evolutionary path than the influenza viruses. In fact, the evolutionary ancestry of ISAV F2 may be more related to the coronavirus (CoV) fusogens. Embecoviruses, such as bovine CoV, human CoV-OC43, and mHV, belong to a subgenus of the beta-coronaviruses that utilizes separate HE and S proteins, similar to the glycoprotein arrangement in ISAV. While the S protein in the embecoviruses is functionally equivalent to ISAV F, the HE is a more recent addition to the beta-coronavirus proteome and has differing involvements in attachment (62, 63). Bovine CoV and mHV HE are involved in binding to *O*-acetylated sialic acids (63–66); however, in human coronavirus OC43, the glycan-binding activity of its HE was progressively lost in adaptation to the human respiratory tract (67). Other beta-coronaviruses, such as SARS-CoV-2, which belongs to a different subgenus (*Sarbecovirus*), do not contain a HE glycoprotein; its attachment and fusion are facilitated both by the S glycoprotein. It has been previously proposed that some beta-coronaviruses, such as mHV, continue to utilize a two-component glycoprotein system with HE and S for virion attachment and fusion, respectively, but during evolution, HE proteins from other CoVs have lost the ability to bind *O*-acetylated sialic acid glycans and instead extended their glycan receptor specificity to the S glycoprotein (65). Lineages B and C contain fusogens from viruses that display distinct viral GPs and membrane fusion mechanisms relative to the other class I viral fusion proteins displayed in the dendrogram. In summary, classification by primary sequence does not capture the functional relationships of the viral GPs, whereas structural classification does.

### Conclusion.

We successfully determined the postfusion structure of an ancient retroviral fusion protein from a PERV. This structure reveals the prototypical 6HB, which is well conserved among class I viral fusion proteins. CD experiments demonstrate that the stability of PERV TM is independent of pH, which is unique relative to other class I viral fusogens that fuse at the plasma membrane. The conserved surface analysis of the PERV TM highlights the importance of the chain reversal and tether regions that undergo significant conformational changes between the pre- and postfusion states. Structure-based phylogenetic analysis of class I viral fusogens uncovered a stratification that corresponds to mechanism of viral entry. Thus, even though all class I viral fusion proteins share a 6HB postfusion core, structural differences at the chain reversal region allow for subclassification that correlates with entry mechanism. The use of structural phylogenetic analysis has potential for use as a tool to qualitatively assign function to poorly characterized viral fusion protein structures and to identify “structural ancestry.”

## MATERIALS AND METHODS

### PERV TM expression and purification.

DNA corresponding to a PERV-A TM (residues 493 to 587; GenPept accession no. AAQ83899) was codon optimized, synthesized, and cloned into a pET-46 Ek/LIC vector (Novagen/MilliporeSigma). A thrombin protease site was inserted after the vector-encoded N-terminal 6-histidine tag, and a single cysteine-to-serine mutation (C557S) was introduced in the CX_6_CC motif via site-directed mutagenesis to prevent nonspecific disulfide-mediated protein aggregation. The PERV TM_493–587_ construct was transformed into SHuffle T7 Escherichia coli cells and grown in 1 L LB media at 37°C. At an optical density at 600 nm of 0.6, the cultures were induced with a final concentration of 0.5 mM isopropyl β-d-1-thiogalactopyranoside (BioShop) and grown at 15°C for 16 h. Cells were pelleted 3,000 × *g* for 20 min at 4°C, and the bacterial pellet was resuspended in lysis buffer (150 mM NaCl, 10 mM Tris-HCl, pH 7.5, 10 mM imidazole, and 3.3 mM Triton X-100). Cell lysis was performed using a cell disruption unit (Constant Systems; TS Series 0.75), and the crude extract was centrifuged at 30,310 × *g* for 45 min at 4°C. The supernatant was filtered through a low-protein-binding 0.22-μm polyethersulfone (PES) membrane filter (LifeGene) and applied to a 3-mL Ni-nitrilotriacetic acid (NTA) affinity chromatography column (Qiagen). The captured His_6_-tagged protein was washed with 10 column volumes (CV) of wash buffer (150 mM NaCl, 10 mM Tris-HCl, pH 7.5, 20 mM imidazole, and 0.1 mM Triton X-100) and eluted with 5 CV wash buffer supplemented with 500 mM imidazole. Thrombin (MilliporeSigma) was added at 1 unit per 1 mg of purified PERV TM in dialysis tubing (molecular weight cutoff [MWCO], 10 kDa) and dialyzed against 1 L of 150 mM NaCl, 10 mM Tris-HCl, pH 7.5, and 0.1 mM Triton X-100 at 4°C for 18 h. Tag-cleaved PERV TM was further purified by size exclusion chromatography using a Superdex 75 10/300 GL (GE Healthcare) equilibrated in 150 mM NaCl, 10 mM Tris-HCl, pH 7.5, and 0.1 mM Triton X-100. Fractions containing the PERV TM were pooled in a 10 mM Tris-HCl, pH 7.5, 150 mM NaCl, 20 mM imidazole, and 0.1 mM Triton X-100 buffer. Protein concentration was determined using absorbance at 280 nm, and purity was monitored by 16% SDS-polyacrylamide gel.

### Molecular weight estimation.

The molecular weight of tag-intact PERV TM was estimated using size exclusion chromatography. A Superdex 75 10/300 GL (GE Healthcare) column equilibrated in 10 mM Tris-HCl, pH 7.5, and 150 mM NaCl was calibrated using approximately 1.25 mg γ-globulin (158,000 Da), bovine serum albumin (66,500 Da), ovalbumin (44,000 Da), carbonic anhydrase (30,000 Da), and myoglobin (17,000 Da). Gel-phase distribution coefficient (*K*_av_) values were determined for each protein standard according to the following equation:

$$K_{\text{av}}=\;(V_{e}-V_{o})/(V_{c}-V_{o})$$

where *V_e_* corresponds to the elution volume, *V_c_* is the total column volume (24 mL), and *V_o_* is the void volume (8 mL based on the γ-globulin elution volume). The calibration curve was generated by plotting *K*_av_ versus log molecular weight of the protein standards. A line of best fit was determined for the calibration curve (*R*^2^ = 0.99), and the equation *K*_av_ = −0.33 × log(MW) + 1.68 was used to estimate the molecular weight of PERV TM (red circle).

### Crystallization trials and structure determination.

Prior to crystallization, purified PERV TM was concentrated to 15 mg · mL^−1^, and a crystal seed stock was prepared by crushing an approximately 100-μm single crystal of murine syncytin A (45% sequence identity) in 50 μL of 24% (wt/vol) polyethylene glycol (PEG) 1500 and 20% (vol/vol) glycerol buffer using a seed bead (Hampton Research). Sitting drop vapor diffusion-based crystallization was performed in 96-well Intelli-Plates (Art Robbins) using a Douglas Instrument Oryx8 liquid handling system. PERV TM postfusion crystals were obtained from a 1:1 ratio of protein to crystallization mother liquor containing 24% (wt/vol) PEG 1500, 20% (vol/vol) glycerol, and 8% (vol/vol) crystal seed stock. All crystallization plates were incubated at 20°C and imaged using the Formulatrix UV Rock Imager 1000 system. Crystals were harvested and directly flash cooled using liquid nitrogen. Diffraction data for the PERV TM crystal were collected at the National Synchrotron Light Source-II (NSLS-II) AMX (17ID-1) beamline. Data were processed using the program DIALS (68) and scaled using AIMLESS (69). The PERV TM structure was determined via molecular replacement using MoRDA in the CCP4 suite (70). A successful molecular replacement solution was identified using the XMRV TM as the search model (PDB ID 4JGS:A). The model was refined in PHENIX.refine (71) and manually built in Coot (72). Model validation was performed using MolProbity (73), Coot (72), and PHENIX.polygon (74). Data collection and refinement statistics are presented in Table 1.

**TABLE 1:** Data collection and refinement statistics for PERV TM (PDB ID 7S94)

Parameter	PERV TM
Data collection	
Wavelength (Å)	0.9201
Beamline	NSLS-II 17ID-1 (AMX)
Space group	P2_1_
Cell dimensions	
a, b, c (Å)	96.9, 34.2, 100.4
α, β, γ (°)	90, 99.9, 90
Resolution range (Å)^*a*^	47.4–2.0 (2.07–2.00)
Total no. of reflections	278,119 (11,949)
No. of unique reflections	41,923 (2,807)
*R*_merge_ (%)^*b*^	16.1 (53.1)
*R*_meas_ (%)^*c*^	17.5 (60.2)
*R*_pim_ (%)^*d*^	6.5 (27.6)
<I/(σ)I>	7.3 (3.1)
CC_1/2_ (%)^*h*^	98.3 (68.4)
Completeness (%)	93.6 (63.9)^*e*^
Redundancy	6.6 (4.3)
Refinement	
No. of molecules in ASU	6
No. of protein atoms	4,030
No. of ligand atoms	34
No. of waters	310
No. of chloride ions	2
*R*_work_/*R*_free_ (%)^*f*^^,^^*g*^	17.5/21.2
Avg. B factor (Å^2^)	
Overall	26.1
Protein	25.6
Solvent	32.1
Ligand	43.6
RMSD bond length (Å)	0.010
RMSD angle (°)	1.06
Ramachandran plot	
Favored (%)	100
Allowed (%)	0
Outliers (%)	0

a Values in parentheses are for the highest-resolution shell.

b *R*_merge_, merging *R* factor = ΣΣ*_j_*|*I_j_* − |/Σ‖, where *I_j_* and represent the diffraction intensity values of the individual measurements and the corresponding mean values, respectively. The summation is over all unique measurements.

c *R*_meas_, multiplicity-independent *R* factor = Σ_hkl_[N_hkl_/(N_hkl_ − 1)]^1/2^ Σ_i_|I_i_(hkl) − <I(hkl)>|/Σ_hkl_Σ_i_I_i_(hkl).

d *R*_pim_, precision-indicating merging *R* factor = Σ_hkl_[1/(N_hkl_ − 1)]^1/2^ Σ_i_|I_i_(hkl) − <I(hkl)>|/Σ_hkl_Σ_i_I_i_(hkl).

e Incomplete data (<70%) in the highest-resolution shell were included to improve electron density map quality and model refinement. Statistics reported reflect the data used to determine the structure that is deposited in PDB.

f *R*_work_ = Σ| |*F*_obs_| − |*F*_calc_| |/Σ|*F*_obs_|, where *F*_calc_ and *F*_obs_ are the calculated and observed structure factor amplitudes, respectively.

g *R*_free_, statistic is the same as *R*_work_ except calculated on 5% of the total reflections chosen randomly and omitted from the refinement.

h CC_1/2_, correlation coefficient between intensities of crystallographic random half data sets.

### Circular dichroism spectroscopy and thermal melts.

Minor modifications were made for the purification of the PERV TM for CD spectroscopy. Briefly, the protein purification process was identical; however, the protein was dialyzed into a buffer containing 10 mM potassium phosphate, pH 7.5, and 150 mM NaF at 4°C overnight at the thrombin digestion stage. The protein was subsequently purified on a Superdex 75 10/300 GL (GE Healthcare/Cytiva) in the same buffer. The CD spectral scans and thermal melting curves for the PERV TM were acquired on a Jasco J-1500 spectropolarimeter in a 1-mm pathlength quartz cuvette (Helma) at a protein concentration of 0.2 mg · mL^−1^. Spectra were recorded over a pH range of 4.5 to 7.5 (pH unit intervals of 0.5) using 10 mM potassium phosphate buffer adjusted to the desired pH. CD wavelength scans were collected at 15°C between 184 and 260 nm at a rate of 50 nm·min^−1^. Raw CD ellipticity values were baseline subtracted, averaged over eight accumulations, and converted to mean residue ellipticity (θ_MRE_) in units of degree cm^2^ · dmol^−1^ as presented in the following equation:

$${\text{θ}}_{\text{MRE}}=\left(\frac{\text{MW}}{N-1}\right).\left(\frac{\theta }{10.d.c}\right)$$

where MW is the molecular weight for each sample in Da, *N* is the number of amino acids, θ is the ellipticity in millidegree, *d* corresponds to the optical pathlength in cm, and *c* is the protein concentration in mg · mL^−1^.

The thermal denaturation assays were carried out at 222 nm, a wavelength selected to maximize the change in CD signal, by increasing the temperature from 15°C to 95°C in 5°C intervals with 120-s equilibration between temperature points. The resultant change in ellipticity was normalized between 0 (folded) and 1 (unfolded) and fit using the program Origin 2017 to a nonlinear biphasic sigmoidal curve to determine the apparent melting temperatures (*T_m_*).

### Retroviral sequence alignment and conserved surface analysis.

Class I retroviral envelope TM protein sequences from bovine leukemia virus (UniProt accession no. Q90M13), feline leukemia virus (UniProt accession no. Q66917), Gibbon ape leukemia virus (UniProt accession no. Q9YWM2), human endogenous retrovirus H (UniProt accession no. Q9N2K0), human endogenous retrovirus V (UniProt accession no. B6SEH8), human T-lymphotropic virus-1 (UniProt accession no. P23064), koala retrovirus (UniProt accession no. Q9TTC0), Mason-Pfizer monkey virus (UniProt accession no. P07575), Moloney murine leukemia virus (UniProt accession no. Q8UMZ9), RD114 retrovirus (UniProt accession no. A7LKA7), simian retrovirus 1 (UniProt accession no. P04027), squirrel monkey retrovirus (UniProt accession no. P21412), avian spleen necrosis virus (UniProt accession no. P31796), syncytin 1 (UniProt accession no. Q9UQF0), syncytin 2 (UniProt accession no. P60508), and xenotropic murine leukemia virus-related virus (UniProt accession no. F2QL75) were selected based upon polybasic furin-like cleavage sites. The amino acid sequences of the TM domains were aligned using Clustal Omega (75). The Clustal Omega output was uploaded to the ConSurf server to map the conserved surface residues onto the PERV TM structure.

### Structural alignment and dendrogram generation.

Class I viral fusion protein structures deposited in PDB with resolutions of 4.5 Å or better and contained an intact HR1, CR, and HR2 were selected for structural alignment. Fusogen structures from avian sarcoma leukosis virus (PDB ID 5H9C), bovine leukemia virus (PDB ID 2XZ3), California Academy of Science virus (PDB ID 4N21), Ebola virus (PDB ID 2EBO), Guanarito virus (PDB ID 4C53), human parainfluenza virus type 3 (PDB ID 1ZTM), human metapneumovirus (PDB ID 5L1X), human T-lymphotropic virus (PDB ID 1MG1), infectious salmon anemia virus (PDB ID 4XYP), influenza A virus (PDB ID 1HTM), influenza B virus (PDB ID 3BT6), Lassa virus (PDB ID 5OMI), lymphocytic choriomeningitis virus (PDB ID 3MKO), Mason-Pfizer monkey virus (PDB ID 4JF3), Marburg virus (PDB ID 4G2K), murine hepatitis virus (PDB ID 6B3O), Newcastle disease virus (PDB ID 3MAW), respiratory syncytial virus (PDB ID 3RRR), severe acute respiratory syndrome coronavirus 2 (PDB ID 6XRA), syncytin 1 (PDB ID 6RX1), syncytin 2 (PDB ID 6RX3), and xenotropic murine leukemia virus-related virus (PDB ID 4JGS) were chosen. Structures were modified in PyMol such that the helical turns N-terminal to the HR1 stutter were removed. The structural alignment between class I viral fusion protein TMs was determined using the DALI Protein Structure Comparison server (76–78). The output Newick tree from DALI was reconstructed using MEGA (79).

### Data availability.

Atomic coordinates and structure factors for the PERV TM structure have been deposited in the PDB under accession no. 7S94.
